# Acknowledgement to Reviewers of *Journal of Fungi* in 2017

**DOI:** 10.3390/jof4010011

**Published:** 2018-01-11

**Authors:** Journal of Fungi Editorial Office

**Affiliations:** MDPI AG, St. Alban-Anlage 66, 4052 Basel, Switzerland

Peer review is an essential part in the publication process, ensuring that Journal of Fungi maintains high quality standards for its published papers. In 2017, a total of 73 papers were published in the journal. Thanks to the cooperation of our reviewers, the median time to first decision was 20 days and the median time to publication was 39 days. The editors would like to express their sincere gratitude to the following reviewers for their time and dedication in 2017:
Abdullah M. S., Al-HatmiLinda, HuangAbhishek, ShrivastavaLindsay, DuttonAdnane, SellamLucinda, BessaAlbina Cristina Ribeiro, FrancoŁukasz, StępieńAleksey, PorolloMan-gi, ChoAlexander, KimMarc Thilo, FiggeAlexandre, AlanioMarc, SwidergallÁlvaro, Rodríguez-GonzálezMaria Helena, FigueiralAmanda F., CurrieMariano Sánchez, CrespoAmir, FarnoudMarie Claude, MenetAna, Espinel-IngroffMark R., BleackleyAndreas H., GrollMark S., GresnigtAngie, GelliMartin, SchmidtAnn, KwanMartine, BertrandAnna, BiernasiukMasoud, Ahmadi-AfzadiAnna, Bzducha-WróbelMelanie, IkehAntoine, LoquetMichael, SamishAntonios, MakrisMichal A., OlszewskiAtsushi, IwataMichela, StaraceAya, YanagawaMira, EdgertonBenjamin L., SchulzNeeraj, ChauhanBhushan, ShresthaNicanor, AustriacoBrian L., WickesNicolina, DiasBrian, MonkOier, EtxebesteChantal, FradinPaula, SampaioCarmen, RomeraloPaula, SundstromCarolina, CoelhoPeter N., LipkeChad, RappleyePetra, PatakovaChangfu, ZhuPhilipp P., BosshardChaoyang, XuePranab, MukerhjeeCharlotte, BerkesRalf, WelschChristian, KubicekRebecca, DrummondChristina C., ChangRebecca, HallChristoph, ZutzRobert L., McFeetersChristophe, BilletteRoderick J., HayDion C., MundyRohitashw, KumarDaniel, EladRosario, SerpicoDaniela, TudorRuiyong, ZhangDarin, WiesnerSalome, Leibundgut-LandmannDavid A., KaufmanSanghyeob, LeeDayvison F. S., FreitasSanjay K., SinghDesa, LilicSara C., RobinsonElena, CasacubertaSarah, KiddElio, CenciSbyren, De. HoogElizabeth, BallouShaobin, ZhongEric, DannaouiShawn R., LockhartFabio Ribeiro, BragaShin-Yi, MarzanoFerry, HagenShu Shun, LiFlorence, FontaineSlavena, VylkovaFrancisco, SolanoSona, KucharikovaFrédéric, LamothSteffen, RuppFusheng, ChenStephane, JeanmartGiorgio, GnaviStephen P., SavilleGiovanna Cristina, VareseStephen, FreeGiovanna, SimonettiSurabhi, NaikGregory M., GauthierSusan G. W., KaminskyjHajime, YaegashiSusan, JarvisHarika, VemulaTakashi, FukudaHerminia, De La VargaTaro, NakamuraHiroyuki, KurokochiTetsuya, HommaInmaculada, Garrido-JuradoTihana, BicanicIsabel Joao, Soares SilvaTimothy, BreakJ. Andrew, AlspaughTomasz, GóralJohn E., HallsworthUlrike, BinderJavier, ArroyoValeria Cizewski, CulottaJean-Michel, SavoieValeria, ScalaJeniel, NettVassileios, VarelasJessie, GlaeserVéronique, EparvierJesus Manuel, CantoralVincent, BrunoJigar, DesaiVishu Kumar, AimaniandaJoanne, LivermoreVito, ValianteJohn, BennettViviane, BalloyJonathan, CummingVolker, SchroeckhJorge, ToledoWalter, BuzinaJos, HoubrakenWilliam H., CarrJosé A. S., MoreiraYohei, TashiroJose Antonio, Reales-CalderónYoshihide, UsamiJosette, PerrierYoshikazu, OhyaJoy, SturtevantYoshimi, AkiraJulia, Garcia-DiazYounes, SmaniKaty C., KaoZenezini Chiozzi, RiccardoKausik, DattaZhe, WangLei, Huang


